# Green Biofabrication of Silver Nanoparticles of Potential Synergistic Activity with Antibacterial and Antifungal Agents against Some Nosocomial Pathogens

**DOI:** 10.3390/microorganisms11040945

**Published:** 2023-04-04

**Authors:** Fatimah O. Al-Otibi, Mohamed Taha Yassin, Abdulaziz A. Al-Askar, Khalid Maniah

**Affiliations:** Botany and Microbiology Department, College of Science, King Saud University, Riyadh 11451, Saudi Arabia

**Keywords:** green synthesis, *Camellia sinensis*, silver nanomaterials, antimicrobial, synergism

## Abstract

Nosocomial bacterial and fungal infections are one of the main causes of high morbidity and mortality worldwide, owing to the high prevalence of multidrug-resistant microbial strains. Hence, the study aims to synthesize, characterize, and investigate the antifungal and antibacterial activity of silver nanoparticles (AgNPs) fabricated using *Camellia sinensis* leaves against nosocomial pathogens. The biogenic AgNPs revealed a small particle diameter of 35.761 ± 3.18 nm based on transmission electron microscope (TEM) graphs and a negative surface charge of −14.1 mV, revealing the repulsive forces between nanoparticles, which in turn indicated their colloidal stability. The disk diffusion assay confirmed that *Escherichia coli* was the most susceptible bacterial strain to the biogenic AgNPs (200 g/disk), while the lowest sensitive strain was found to be the *Acinetobacter baumannii* strain with relative inhibition zones of 36.14 ± 0.67 and 21.04 ± 0.19 mm, respectively. On the other hand, the biogenic AgNPs (200 µg/disk) exposed antifungal efficacy against *Candida albicans* strain with a relative inhibition zone of 18.16 ± 0.14 mm in diameter. The biogenic AgNPs exposed synergistic activity with both tigecycline and clotrimazole against *A. baumannii* and *C. albicans*, respectively. In conclusion, the biogenic AgNPs demonstrated distinct physicochemical properties and potential synergistic bioactivity with tigecycline, linezolid, and clotrimazole against gram-negative, gram-positive, and fungal strains, respectively. This is paving the way for the development of effective antimicrobial combinations for the effective management of nosocomial pathogens in intensive care units (ICUs) and health care settings.

## 1. Introduction

Nosocomial microbial infections represent a public health burden worldwide, contributing to a high number of morbidities and fatalities every year [1]. These infections refer to illnesses that have occurred among hospitalized patients and are the result of various toxins or infectious agents [2]. There is a wide range of variance in the rates of occurrence of nosocomial infections across different geographical areas and during different periods of time [3]. Nosocomial bloodstream infection (NBSI), nosocomial pneumonia infection (NPNEU), nosocomial surgical site infection (SSI), and nosocomial urinary tract infection (UTI) account for 80% of all nosocomial infections [4]. The main cause behind the incidence of nosocomial infections is drug-resistant strains [5]. In developing and developed countries, it is projected that 10 and 7 patients out of every 100 inpatients in healthcare facilities may become infected with hospital-acquired illnesses, respectively [6]. In this context, several microbial strains, including methicillin-resistant *Staphylococcus aureus* [7], *Escherichia coli* [8], *Acinetobacter baumannii* [9], and *Candida albicans* [10], have been reported to cause hospital-acquired infections. Due to their role in a number of infectious diseases that can be fatal, multidrug-resistant bacteria pose a severe threat to public health globally [11]. Pollution, mutation, changing environmental circumstances, and excessive drug usage all contribute to the ongoing rise in multidrug-resistant bacterial species [12,13]. To solve this issue, researchers are working to create novel medications for the treatment of these microbial illnesses [14].

Nanotechnology provides a distinct platform for the modification and production of unique metal features ranging in size from 1–100 nm with potential applications in cell labeling, diagnostics, biomarkers, pharmaceutics, mechanics, optics, agriculture, electronics, biomedicine, and antimicrobial applications [15,16,17,18]. The AgNPs have a large surface area and small particle size, which allows for extensive contact with microorganisms [19,20]. These characteristics of AgNPs significantly enhance their biological and chemical capabilities, which notably aid in their demonstration as potent bactericidal compounds [21]. Nanometals were distinguished by their oligodynamic impact, which was the microbicidal action of metals, particularly heavy metals, at low concentrations [22]. In this respect, silver is one of the oligodynamic materials that has received a lot of attention because of its low toxicity, high efficacy, and broad variety of disinfection uses [23]. The high surface area of AgNPs is the major distinguishing feature of oligodynamic materials, enabling them to bind effectively to bacterial cells, penetrating them, and producing free radicals and reactive oxygen species [24]. Briefly, AgNPs have four recognized antibacterial effects: (1) adherence to the microbial membrane’s surface; (2) when AgNPs enter cells, they disrupt biomolecules and cause intracellular damage; (3) they cause cellular toxicity by producing ROS that create oxidative stress in the cell; and (4) they interfere with the cell’s signal transduction pathways [25].

The green synthesis of AgNPs via biological methods offers numerous benefits over physical and chemical synthesis since these methods use hazardous materials, have high equipment costs, and generate toxic compounds, while biological synthesis is simple, inexpensive, non-toxic, and environmentally friendly [26]. Due to the availability of several plants and their straightforward and secure application, plant-mediated synthesis of AgNPs is an extensively used approach for the biofabrication of AgNPs [27,28]. The facile bioformulation of silver nanomaterials is mediated by the presence of many phytochemical ingredients in plant extracts, such as flavonoids, phenols, tannins, polysaccharides, amino acids, terpenoids, and proteins [29]. For instance, the aqueous extracts of *Zataria multiflora*, *Ctenolepis garcini* leaves, and *Moringa oleifera* seeds mediated the green bioformulation of AgNPs against different bacterial strains such as *S. aureus*, *Pseudomonas aeruginosa*, *E. coli*, and *Salmonella typhi*, recording inhibitory zones ranging from 14 to 28.5 mm [30,31,32]. Recent research found that biogenic AgNPs had synergistic action with fluconazole and metronidazole antifungals against *C. albicans*, as well as synergistic activity with ciprofloxacin, cefixime, and bacitracin antibiotics against *P. aeruginosa* strains [33]. Several reports investigated the synergistic potency of AgNPs with antibiotics such as streptomycin, amikacin, ampicillin, and polymyxin B against *E. coli*, *S. aureus*, and *A. baumannii* [34,35,36].

Green tea extract contains a high level of polyphenolic compounds such as catechol, caffeine, 1,2,3-benzenetriol, 1,3,5-benzenetriol, and methoxy resorcinol, indicating the possibility of production of AgNPs utilizing this extract since phenolic components are significant in metal reduction [37]. Previous research detailed the green synthesis of AgNPs using green tea extract and proved the antibacterial activity of the biogenic AgNPs against *Klebsiella* spp. and *S. aureus* at doses ranging from 5 to 20 mg/mL [38]. Another study used green tea extract to create AgNPs and showed antibacterial efficacy against *E. coli* and *S. aureus*, with inhibition zones of 16 and 14 mm, respectively [39]. Previous studies have confirmed the possible biosynthesis of AgNPs using green tea extract due to its high polyphenolic component and antioxidant content; however, none of the prior studies have looked into the synergistic patterns of AgNPs with antibacterial and antifungal drugs. As a result, the present study was designed to evaluate the synergistic effects of AgNPs produced utilizing green tea extract with antimicrobial drugs against some nosocomial bacterial and fungal strains.

## 2. Materials and Methods

### 2.1. Preparation of Green Tea Leaves Extract

The Chinese green tea, namely *Camellia sinensis* var. *sinensis,* which is characterized by its small leaves, was obtained from a local market in Saudi Arabia. The plant materials were identified by the Botany and Microbiology Department’s Herbarium Staff and deposited in the herbarium with voucher number KSU-23592. For the preparation of the water extract of *C. sinensis* leaves, the leaves were first washed with running tap water, then rinsed in distilled water, and then dried completely. Afterward, the dried leaves were blended using a mechanical mortar to obtain the homogenous powder, and then 50 g of the powdered leaves were placed in an Elementyer flask (500 mL) for submerging in 200 mL distilled water under heating at 70 °C for half an hour. The aqueous extract of green tea leaves was incubated over a magnetic stirrer for 24 h at 25 °C, then filtered using Whatman filter paper (1) to get rid of any impurities [40,41,42]. Preservation of the extract in the refrigerator was conducted for subsequent AgNP synthesis.

### 2.2. Green Biofabrication of AgNPs

The leaf water extract of *C. sinensis* was utilized as a reducing agent for the formulation of AgNPs using silver nitrate solution. The salt of AgNO_3_ was purchased from Sigma-Aldrich, Saint Louis, MO, USA. Ten milliliters of the green tea extract were added to 90 mL of AgNO_3_ solution (1 mM) and then incubated in a shaking incubator under dark conditions for 24 h at 25 °C. The visual observation of the color change of the colorless silver nitrate solution to dark brown following incubation with green tea extract initially verified the formation of AgNPs. For harvesting the biogenic AgNPs, the reaction mixture was centrifuged at 10,000 rpm for 10 min, and the supernatant was discarded. The collected precipitate was washed thrice with distilled water for removal of any remaining extract, then incubated at 80 °C for complete drying and subsequent analytical characterization.

### 2.3. Physicochemical Characterization of Biogenic AgNPs

The optical absorption of the biogenic AgNPs was conducted using a UV-Vis spectrophotometer (UV-1601, Shimadzu, Kyoto, Japan), while Fourier transform infrared spectroscopy (FTIR) measurements were performed to investigate the interaction between plant metabolites and the biosynthesized AgNPs. In addition, the morphological features of the biogenic AgNPs were studied using transmission electron microscope (JEOL, JEM1011, Tokyo, Japan) analysis for the investigation of particle shape, size, and distribution. The elemental pattern of the biogenic AgNPs was examined using energy dispersive X-ray (EDX) analysis (JEOL, JSM-6380 LA, Tokyo, Japan). Moreover, the crystallographic characteristics were studied using X-ray powder diffraction (XRD) examination (Shimadzu, Columbia, MD, USA), whereas the surface charge and hydrodynamic diameter of the fabricated AgNPs were detected using a Zeta sizer instrument (Malvern Instruments Ltd.; zs90, Worcestershire, UK) [43,44].

### 2.4. Antimicrobial Potency of Biogenic AgNPs against Different Microbial Strains

Four nosocomial microbial strains were investigated for their susceptibility to the biogenic AgNPs, namely *C. albicans* (ATCC 18804), MRSA (ATCC 33592), *A. baumannii* (ATCC 43498), and *E. coli* (ATCC 25922). The microbial suspension was prepared using sterile saline solution (0.85%) by picking up the colonies of 24 h cultures and dipping them into the saline solution [40,41]. The turbidity of the microbial suspension was adjusted using 0.5 McFarland standard to achieve viable bacterial and fungal cell counts of 1.0 × 10^8^ and 1.0 × 10^6^ cfu/mL, respectively. The prepared microbial suspensions (0.5 mL) were pipetted and spread over freshly prepared Mueller–Hinton agar (MHA) medium. Tigecycline and clotrimazole, as standard antibacterial and antifungal agents, respectively, were purchased from Sigma–Aldrich (St. Louis, MO, USA). The antimicrobial agents were solubilized in methanol and sonicated to ensure complete solubilization, then sterile filter paper disks were impregnated with tigecycline and clotrimazole as antibacterial and antifungal positive controls to achieve final concentrations of 15 and 10 μg/disk, respectively. In addition, sterile filter paper disks (8 mm in diameter) were loaded with 200 μg of AgNPs after their solubilization in methanol, while filter paper disks filled with methanol only were used as negative controls. Finally, the loaded disks were placed over the seeded layer and then preserved for 4 h in the refrigerator to allow AgNPs diffusion. The inhibition zones were measured using a Vernier caliper after a 24-h incubation period at 35 °C. The minimum inhibitory concentration of biogenic AgNPs generated from *C. sinensis* leaf extract was determined using a broth microdilution assay in 96-well microtitre plates against *E. coli* and *C. albicans* strains [45,46]. In addition, the minimum bactericidal (MBC) and fungicidal (MFC) concentrations were evaluated by culturing inoculums from MIC wells over freshly prepared MHA plates, incubating them at 35 °C overnight, and finally examining the plates for bacterial and fungal growth. The lowest concentrations of AgNPs that showed no bacterial or fungal growth were registered as MBC and MFC, respectively.

### 2.5. Investigation of the Ultrastructural Changes of Microbial Cells Treated with AgNPs Using SEM Analysis

The deformations caused by the biogenic AgNPs against MRSA, a gram-positive strain; *E. coli*, a gram-negative strain; and also against the fungal strain, *C. albicans*, were observed by scanning electron microscopy (SEM) examination. Agar pieces were taken from the inhibition zones and preserved in 3% (*v*/*v*) glutaraldehyde for 1 h at 25 °C (buffered with 0.1 M sodium phosphate buffer, pH 7.2). The fixed agar pieces were then washed four times in buffer. The pieces were washed four times in buffer after being post-fixed in 1% (*w*/*v*) osmium tetroxide (OsO4) for 1 h. The samples were then alcohol dehydrated for 15 min using a variety of ethanol strengths (30–100%). The samples were dried before being adhered to stubs using double-sided carbon tape. A Polaron SC 502 sputter coater was then used to deposit a thin layer of gold onto the specimens. Lastly, they were investigated using a scanning electron microscope (JEOL JSM-6380 LA).

### 2.6. Synergistic Antimicrobial Efficiency of Biogenic AgNPs with Antimicrobial Agents

The MIC concentration of biogenic AgNPs was tested for synergistic activity with tigecycline and clotrimazole antimicrobial agents against the tested bacterial and fungal pathogens using the standard disk diffusion assay [47]. In addition, the synergism of AgNPs with linezolid was also assessed against MRSA strain. For this, sterile filter paper disks were loaded with MIC and two-fold MIC concentrations of AgNPs, while another group of disks was impregnated with both AgNPs (40 μg/disk) and tigecycline (15 μg/disk). In addition, filter paper disks loaded with methanol were utilized as a negative control, whereas tigecycline disks were used as positive controls. Moreover, clotrimazole (10 μg/disk) and linezolid (30 μg/disk) disks were tested for synergism with AgNPs against *C. albicans* and MRSA strains, respectively. The impregnated disks were placed over the MHA plates seeded with microbial suspensions, as mentioned above. Finally, the plates were incubated at 35 °C overnight, and then inhibition zones were measured for determination of relative synergistic activity as demonstrated in the following equation:$\;synergism\;\%=\frac{B-A}{A}\times 100$, whereas A and B are the inhibition zones for the standard antimicrobial agent and antimicrobial agent+ AgNPs, respectively. The increase in fold of inhibition area (IFA) was calculated according to the following equation [48]:

(IFA) = (B^2^ − A^2^)/A^2^

The fractional inhibitory concentration (FIC) index was calculated according to the following formula as presented in earlier studies [49,50].

FIC index (FICI) = $\frac{\mathrm{MIC}\;\mathrm{of}\;\mathrm{drug}\;A\;\mathrm{in}\;\mathrm{the}\;\mathrm{combination}}{\mathrm{MIC}\;\left(A\right)}+\frac{\mathrm{MIC}\;\mathrm{of}\;\mathrm{drug}\;B\;\mathrm{in}\;\mathrm{the}\;\mathrm{combination}}{\mathrm{MIC}\;\left(B\right)}$, whereas A referred to AgNPs and B referred to antibiotics.

The FICI detected as follows: FICI ≤ 0.5 synergistic effect, FICI = 0.5 to 1 additive effect, 2 ≤ FICI < 4 indifferent, and 4 <FICI antagonism.

### 2.7. Statistical Analysis

The data of current investigation was analyzed using GraphPad Prism version 8.0 (GraphPad Software, Inc., La Jolla, CA, USA) via one-way analysis of variance and Tukey’s test. All experimentations were done in triplicates, and the data were presented as the mean of triplicates ± standard error.

## 3. Results and Discussion

### 3.1. Green Biosynthesis of AgNPs

**Figure 1:**
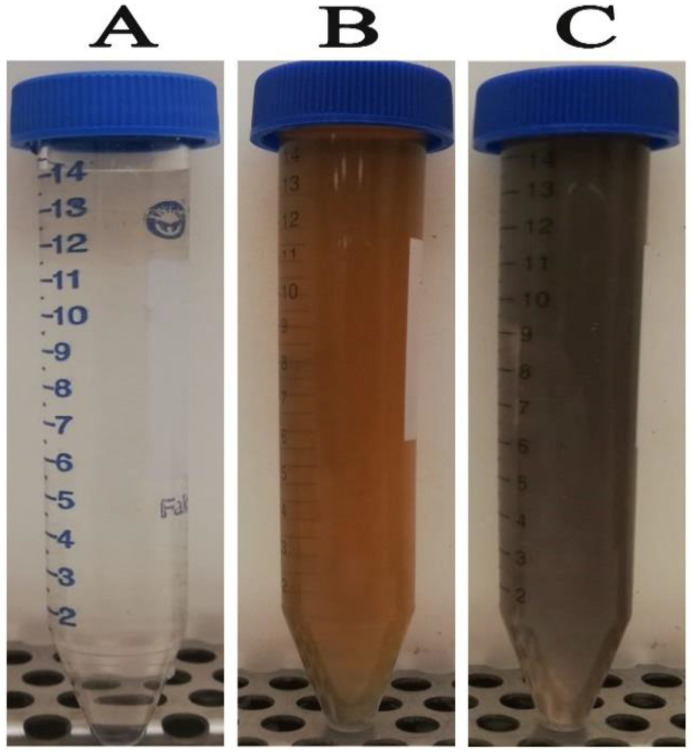
Green bioformulation of AgNPs using green tea extract. (**A**) refers to AgNO_3_ solution, (**B**) refers to water extract of green tea leaves, and (**C**) biogenic AgNPs.

**Figure 2:**
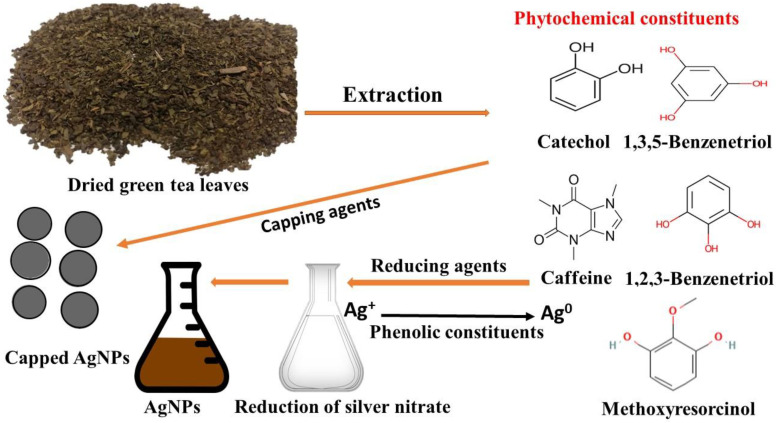
Schematic illustration of the green synthesis of biogenic silver nanoparticles (AgNPs) utilizing green tea leaves.

**Figure 3:**
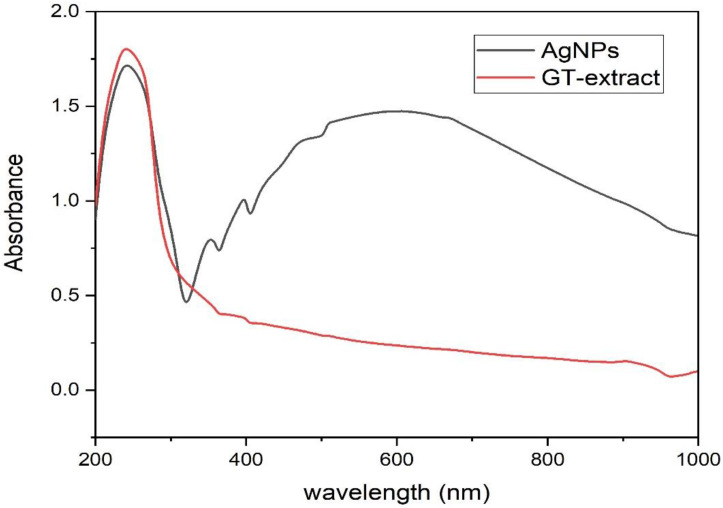
UV spectrum of the biogenic silver nanoparticles (AgNPs) and green tea (GT) water extract.

The primary indication of silver nanoparticle formation is the observed color change of the colorless silver nitrate solution after the addition of the plant extract. Figure 1A shows the colorless silver nitrate solution that, when reduced by green tea extract (Figure 1B), produced AgNPs with a dark brown color (Figure 1C). In this setting, the color change indicated the reduction of Ag^+^ ions through the action of plant metabolites leading to the formation of elemental nanosilver. Previous reports indicated that catechol, caffeine, 1,3,5-benzenetriol, 1,2,3-benzenetriol, and methoxy resorcinol, were the main phytoconstituents of aqueous leaf extract of *C. sinensis*, contributing to the reduction, stabilizing and capping of AgNPs (Figure 2) [51]. The detected UV peak at λ_max_ around 200 nm could be allocated to the adsorption of organic molecules on the AgNPs surface during the reduction process as phenolic acids, flavonoids, and heteroatoms such as S, O, N, and unsaturated groups [52]. The broad absorption peak noticed at 600 nm could be assigned to the surface plasmon resonance (SPR) of the biogenic AgNPs (Figure 3). The SPR peak of biogenic AgNPs has shifted to a longer wavelength at 600 nm due to the aggregation of AgNPs [53,54].

### 3.2. TEM Analysis of the Biofabricated AgNPs

**Figure 4:**
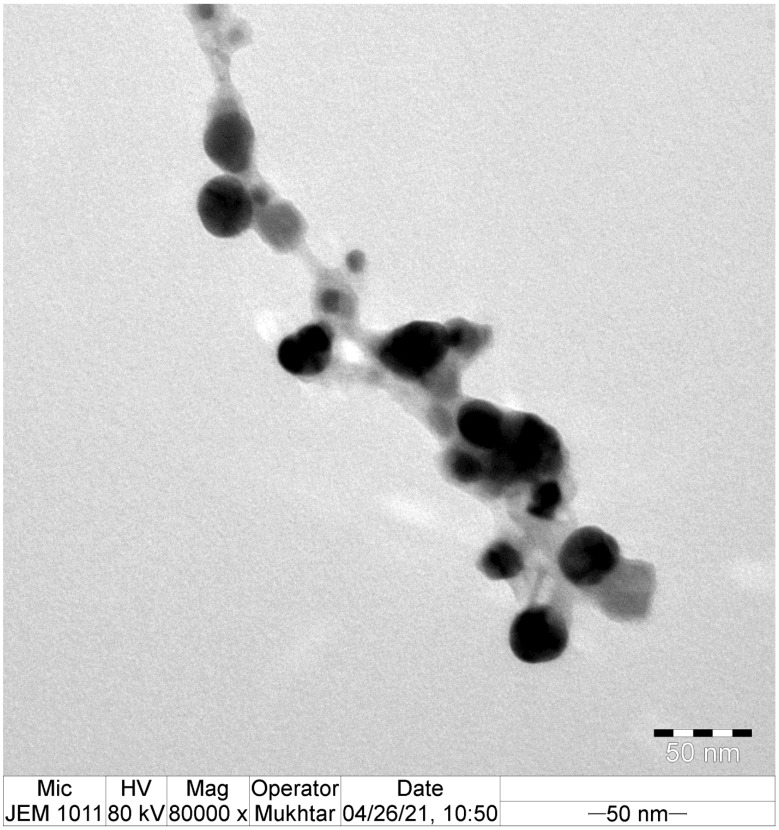
TEM micrograph of the eco-friendly synthesized AgNPs.

**Figure 5:**
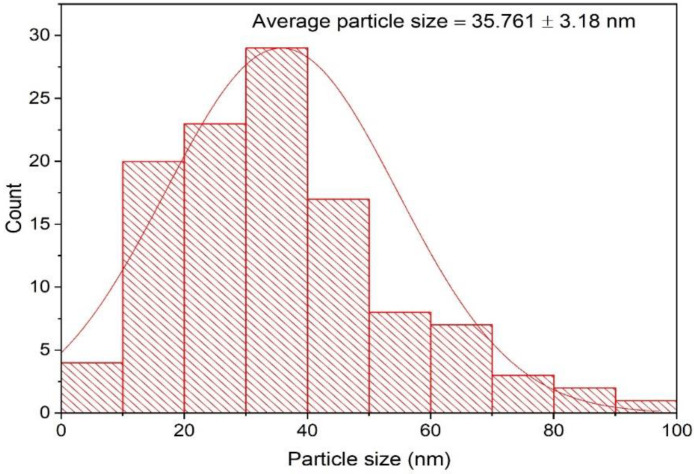
Particle size distribution histogram of the bioformulated AgNPs.

The morphological features of the eco-friendly synthesized AgNPs of green tea extract were investigated using TEM analysis. TEM micrographs indicated that the bioformulated AgNPs were spherical in shape with the presence of capping biomolecules, as seen in Figure 4. The average particle size of the biosynthesized AgNPs was detected to be 35.761 ± 3.18 nm, as demonstrated in Figure 5. Our findings were in accordance with those of a previous investigation, which indicated that the average particle size diameter of green tea-fabricated AgNPs was 34.68 ± 4.95 nm [55].

### 3.3. Elemental Investigation of the Biofabricated AgNPs

**Figure 6:**
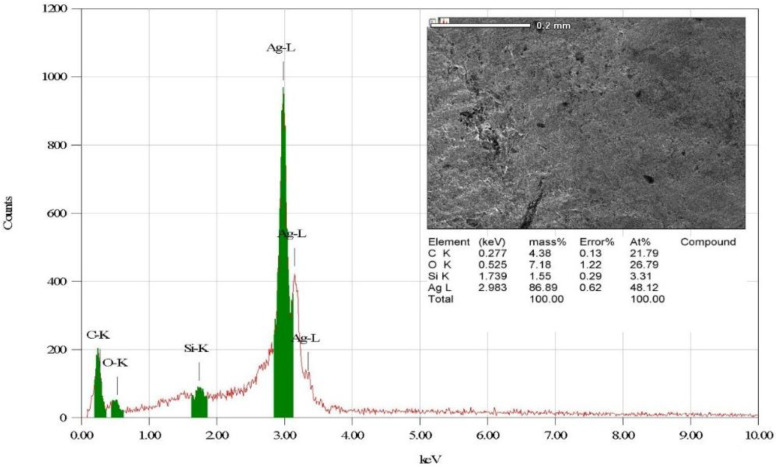
Elemental configuration of the bioformulated AgNPs.

The elemental composition of the eco-friendly synthesized AgNPs was investigated utilizing energy-dispersive X-ray (EDX) analysis. EDX investigation indicated that the elemental mapping of the biogenic AgNPs was found to be composed of the following elements: silver, oxygen, carbon, and silicon Figure 6 [56]. The strong peak found at 2.983 keV was assigned to the elemental silver, indicating the successful biofabrication of AgNPs. In addition, the detected peak of elemental carbon was assigned to the carbon tape used for placing the biogenic AgNPs on the sample holder [57]. In addition, a previous investigation explained the presence of weak signals of other elements, such as silicon, in the EDX pattern and related this to the capping agents of the plant extracts adsorbed over the surface of AgNPs [58]. Additionally, the weak silicon peak might be attributable to X-ray emissions from carbohydrates or proteins in the extract [59].

### 3.4. Fourier Transform Infrared Spectroscopy (FT-IR) Investigation of AgNPs

**Figure 7:**
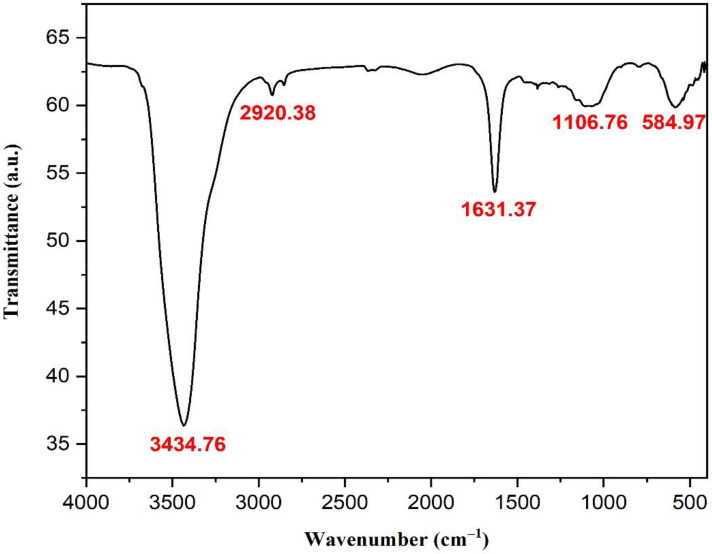
FTIR analysis of the biofabricated AgNPs.

FTIR analysis was used to investigate the bioformulated AgNPs and identify the key functional groups that led to their reduction, capping, and stabilization. In this context, the spectrum of FTIR investigation indicated the presence of five distinctive absorption bands, as seen in Figure 7. The strong band at 3434.76 cm^−1^ was assigned to the O-H stretching of phenolic compounds in *C. sinensis* leaf extract, while the absorption band noticed at 2920.38 cm^−1^ was attributed to C–H stretching of aldehydes from plant metabolites (Table 1) [60,61]. In addition, the sharp peak at 1631.37 cm^−1^ was clearly attributed to the C–O stretching or C–N bending of carboxylic compounds or amides, respectively [62]. Furthermore, the band noticed at 1106.76 cm^−1^ could be assigned to the C–N stretching of aliphatic amines, which clarified the proteins of plant metabolites in the capping of the biosynthesized AgNPs, contributing to their stability and preventing their agglomeration [63]. The peak at 584.97 cm^−1^ could be assigned to the Ag-O stretching, as reported in previous reports [64,65]. Together, the previous findings supported the notion that the functional groups of AgNPs, such as alcohols, phenols, aldehydes, carboxylic compounds, amides, and amines, helped reduce, stabilize, and cap the biofabricated AgNPs, thereby enhancing their stability and preventing agglomeration.

### 3.5. X-ray Powder Diffraction (XRD) Analysis of the Biogenic AgNPs

**Figure 8:**
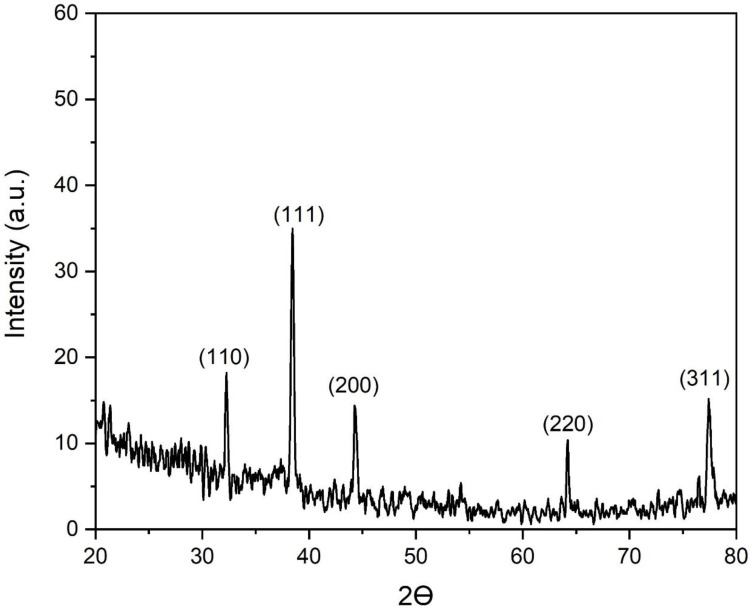
XRD pattern of silver nanoparticles formulated using green tea leaf extract.

The crystallographic properties of the biogenic AgNPs were studied using XRD analysis. In this setting, the XRD pattern revealed the presence of five sharp diffraction peaks at two theta (Ɵ) angles of 32, 38, 44, 64, and 77°, which corresponded to the silver crystal planes of (110), (111), (200), (220), and (311) respectively, as shown in Figure 8. These findings affirmed the face-centered cubic (*fcc*) structure of the biosynthesized AgNPs as indicated by the Joint Committee on Powder Diffraction Standards (JCPDS), file no. 04-0783 [66]. Previous investigations documented the bioformulation of the *fcc* structure of crystalline AgNPs formulated using different biogenic sources, such as *Euphrasia officinalis* leaf extract [67], tuber extract of *Arisaema flavum* [68], the leaf extract of *Holoptelea integrifolia* [69], and *Mangifera indica* leaf extract [70].

### 3.6. Zeta Analysis of the Fabricated Silver Nanomaterials

**Figure 9:**
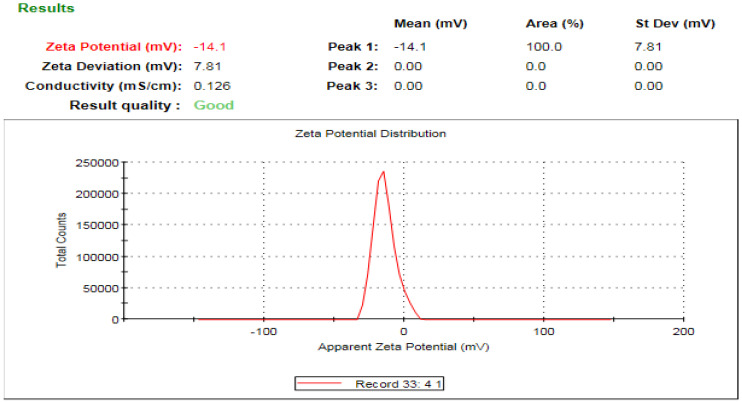
Surface charge of the biogenic silver nanomaterials.

**Figure 10:**
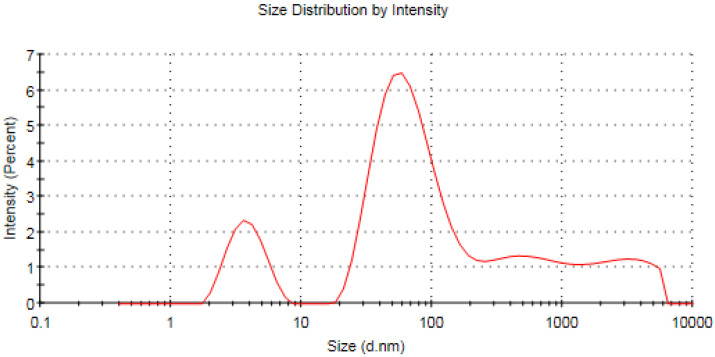
Dynamic light scattering pattern of the bioformulated AgNPs.

The zeta potential is an important indicator of the stability of colloidal dispersion and also a sign of particle-related repulsion or attraction intensity [71]. Zeta potential analysis of the biofabricated AgNPs was conducted to detect the surface charge of the synthesized materials, which was found to be −14.1 mV, as seen in Figure 9. Moreover, the estimated negative charge of the biogenic AgNPs provides colloidal stability owing to the mutual repulsion between the formulated nanomaterials [72]. Furthermore, the estimated negative charge of the synthesized AgNPs was in agreement with that of previous reports indicating the negative charge of biogenic silver nanomaterials synthesized using *Caesalpinia digyna* [73] and *Syzygium aromaticum* extracts [74]. The estimated negative charge on the surface of biogenic AgNPs could be assigned to the action of some capping molecules in green tea extract as the hydroxyl functional group of polyphenolic compounds [75]. On the other hand, the average hydrodynamic diameter of the synthesized nanomaterials was estimated utilizing the dynamic light scattering technique, as seen in Figure 10 [76]. In this context, the average hydrodynamic diameter was estimated to be 42.61 nm, which was slightly higher than that of TEM micrographs owing to the action of capping agents, which formed the core shell structure, in addition to the fact that the DLS technique estimated both the diameters of the nanoparticles and the adjacent hydrate layers [77,78]. The DLS pattern revealed the aggregation of AgNPs, revealing a wide peak in size, ranging from 100 to 1000 nm, which affirmed the action of capping molecules of the plant extract in acting as a shell for the nanoparticle aggregates. That explains the wide peak found in the DLS pattern and also the wide peak found in the UV spectrum around 600 nm [79]. The biogenic AgNPs displayed a PDI value of 0.679, which indicated that the particles might aggregate with time [80].

### 3.7. Screening of Antimicrobial Effectiveness of Biogenic AgNPs

The antibacterial effectiveness of the biogenic AgNPs was tested against MRSA, *E. coli*, *A. baumannii*, and *C. albicans* strains. The biofabricated AgNPs showed anticandidal efficacy against the *C. albicans* strain, with an average particle size diameter of 18.16 ± 0.14 mm and a concentration of 200 μg/disk (Table 2). Silver nanoparticles have remarkable anticandidal efficacy against *C. albicans* by damaging cell membranes and inhibiting normal cell division [81]. In addition, silver nanoparticles’ anticandidal efficiency is caused by the creation of insoluble compounds containing sulfhydryl groups in candidal cell walls, as well as the breakdown of membrane-bound enzymes and lipids, which causes cell lysis [82]. Furthermore, the disruption of the candidal cell membrane and cell wall leads to the destabilization of membrane permeability, resulting in the leakage of potassium ions (K^+^) [83]. These events, triggered by the action of silver nanoparticles, resulting in the initiation of candidal cell death by disturbing cellular permeability, disrupting DNA replication, and inhibiting protein synthesis [84]. Excitingly, the biogenic AgNPs (200 μg/disk) exposed antibacterial bioactivity against the tested bacterial strains, with inhibitory zones of 28.17 ± 0.21, 21.04 ± 0.19, and 36.14 ± 0.67 mm against MRSA, *A. baumannii*, and *E. coli* strains, respectively. The antibacterial potency of the bioformulated AgNPs was significantly higher than that of the tigecycline control (*p* < 0.05), whereas the bioactivity of these nanomaterials against *A. baumannii* strain was not significantly different (*p* > 0.05) compared to control. In this context, the biosynthesized AgNPs have their antibacterial mode of action via a variety of pathways, including the continual discharge of silver ions (Ag ^+^ ions), which is thought to be a microbe killing mechanism, or by the efficacy of AgNPs themselves [85]. The advantage of AgNPs over metallic silver or its salts is the regulated, steady release of Ag^+^ ions, which have a longer-lasting antibacterial impact [86]. Compared to antibiotics, microbes are considerably less likely to develop resistance to AgNPs [87]. The antibacterial efficiency of AgNPs can be explained by four main pathways: the production of reactive oxygen species (ROS) as a result of redox reactions [88]; adherence of AgNPs to the membranes of bacterial cells [89]; disruption of the membranes [90]; intercalation of AgNPs between DNA bases resulting in blockage of DNA replication and transcription [91]; and accordingly, the disruption of ribosomes, which prevents protein synthesis [92]. In addition, the bacterial cell grieved severe damage as a result of the discharge of internal metabolites triggered by the nanoparticle’s passage through the cell membrane, which generated the antibacterial action [93]. The MIC concentration of biogenic AgNPs against *E. coli* strain was found to be 40 μg/disk, while it was detected to be 20 μg/disk against *C. albicans* strain. The MIC concentration of biogenic AgNPs was tested for the synergistic activity with tigecycline, linezolid, and clotrimazole antimicrobial agents against the tested strains. The MBC of the biogenic AgNPs was found to be 80 μg/disk against *E. coli* strain, whereas MFC was detected to be 40 μg/disk against *C. albicans* strain.

### 3.8. Investigation of the Ultrastructural Changes of Microbial Cells Treated with AgNPs Using SEM Analysis

**Figure 11:**
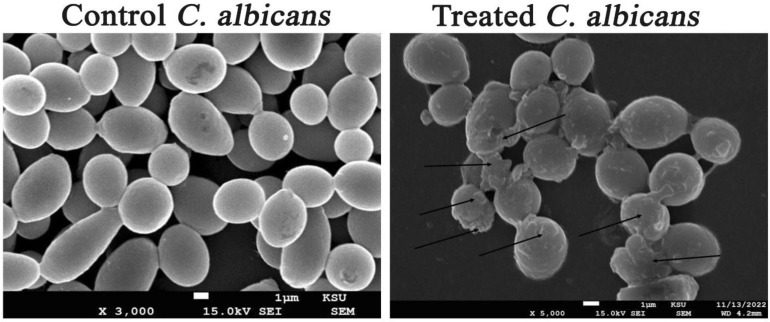
Ultrastructural deformations of *C. albicans* treated with AgNPs.

**Figure 12:**
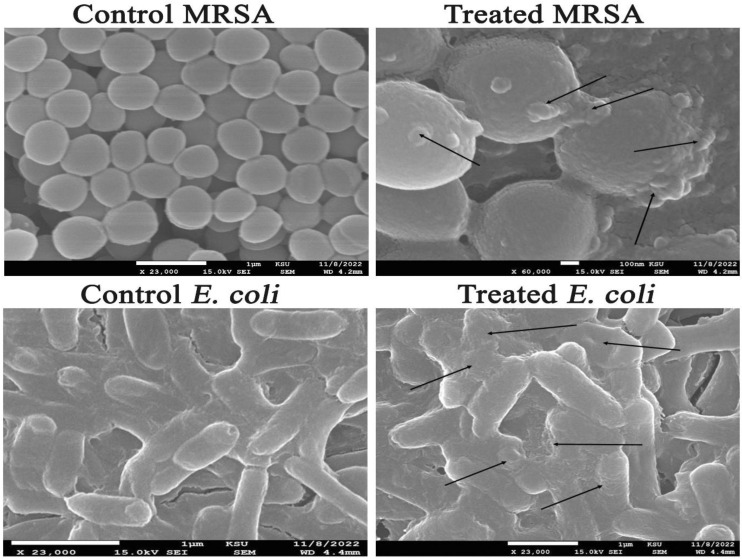
Ultrastructural deformations of MRSA and *E. coli* strains treated with AgNPs.

Figure 11 showed both untreated and AgNP-treated *C. albicans* cells. The treated candidal cells showed distorted characteristics, such as enlarged cells with shriveled cell walls. Furthermore, the SEM investigation showed the deformations of the treated candidal cells with the appearance of cellular remains resulting from the cell wall and cell membrane disruption and, consequently, cell lysis. In addition, SEM examination of AgNPs-treated MRSA cells showed that these cells had an irregular cell shape, with pits and bulges on the cell surface. This might be due to AgNP accumulation over the bacterial cell wall, causing its disruption and the leakage of the cellular constituents and, ultimately, cell death (Figure 12). The control MRSA cells, on the other hand, showed no indications of deformation and were spherical and undamaged. Additionally, the AgNP-treated *E. coli* cells showed signs of damage, including a cracked and wrinkled outer surface and totally warped cell membranes, in contrast to the unaffected control cells, which remained intact. The disruption of cell membranes might be due to AgNP accretion in the bacterial membrane, which causes a shift in membrane potential. As a result, a shift in membrane potential encourages pit development in the membrane and additional cell lysis, ultimately leading to cell death [94].

### 3.9. Synergistic Patterns of AgNPs with Commercial Antimicrobial Agents

**Figure 13:**
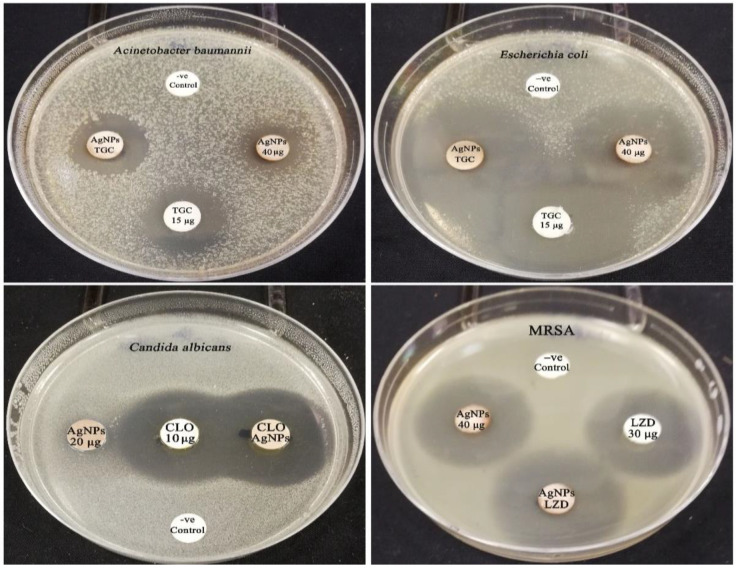
Antimicrobial activity of the biogenic AgNPs and commercial antimicrobial agents (LZD: linezolid, TGC: tigecycline, CLO: clotrimazole).

The synergistic activity of the biogenic AgNPs with commercial antimicrobial drugs was evaluated. According to the results of the synergetic activity assay, the biofabricated AgNPs (40 µg/disk) demonstrated synergistic efficiency with the antibiotic tigecycline against the strains of *A. baumannii* and *E. coli*, with relative synergism percentages of 8.93 and 15.72%, respectively (Figure 13). In contrast, no effect was detected between AgNPs and tigecycline antibiotic against MRSA strain. The inhibitory zone of the antibiotic tigecycline against MRSA strain was found to be 20.56 ± 0.23 mm, whereas the inhibition zone of the combination of AgNPs and tigecycline was found to be 19.89 ± 0.31 mm. As a result, the synergistic action was tested with another commercial antibacterial drug, namely, linezolid. According to Table 3, the combined action of linezolid and AgNPs revealed synergistic activity with a percentage of 12.26%. On the other hand, the synergistic antifungal efficacy of AgNPs with clotrimazole antifungal agent was assessed against *C. albicans* strain, and the results showed that the biofabricated AgNPs (20 µg/disk) exposed synergistic potency, which recorded a relative percentage of 8.58%. In addition, the zone of inhibition of tigecycline was found to increase significantly in the synergism test of *E. coli* plates compared to the initial screening test, which recorded relative inhibitory zones of 35.24 ± 1.49 and 22.67 ± 0.18 mm, respectively, demonstrating the potent synergistic activity of AgNPs with tigecycline even at MIC concentration against *E. coli* strain. The synergistic patterns were found to be low, and the synergism was tested between the MIC concentration of silver nanoparticles and different antimicrobial agents. Hence, we tested the synergistic percentages of two-fold MIC concentration of AgNPs with different antimicrobial agents against *E. coli*, *A. baumannii*, MRSA, and *C. albicans* strains. The two-fold MIC concentration of AgNPs revealed relative synergistic percentages of 48.14, 31.60, 29.70, and 39.30% with different antimicrobial agents against *A. baumannii*, *E. coli*, MRSA, and *C. albicans* strains, respectively. Consequently, the findings supported that the relative synergistic activity was increasing with increasing the concentration of the biogenic AgNPs against the tested strains. The highest synergistic action was seen for AgNPs (2-fold MIC) and tigecycline combination against *A. baumannii*, with an IFA of 1.2, whereas the lowest synergism was observed for AgNPs (two-fold MIC) and linezolid combination, with an IFA of 0.7. Nevertheless, weak synergism was identified between AgNPs (MIC) and tigecycline against *A. baumannii* and *E. coli*, with an IFA value of 0.2, while AgNPs (MIC) and linezolid combination showed an IFA value of 0.3 against MRSA strain. The IFA value increased from 0.2 to 0.9 for AgNPs (MIC)-clotrimazole and AgNPs (two-fold MIC)-clotrimazole combinations, respectively. The fractional inhibitory concentration (FIC) index was calculated to detect the synergistic, additive, or antagonistic effect of the biogenic AgNPs (Table 4). The results of FIC revealed the synergistic activity of AgNPs+ tigecycline combination against *A. baumannii* strain, whereas an additive effect was found for both AgNPs+ linezolid and AgNPs+ tigecycline combination against MRSA and *E. coli* strains with FICI value of 0.38, 0.86, and 0.63 respectively. On the other hand, no effect was detected for AgNPs+ tigecycline combination against the MRSA strain with an FICI value of 1.75. Antifungal synergism of AgNPs+ clotrimazole combination against *C. albicans* strain was detected, recording an FICI value of 0.25.

The synergistic and additive activities of the biogenic AgNPs with tigecycline antibiotic against both gram-negative strains, *A. baumannii* and *E. coli*, were detected, while no effect of this combination was registered against the MRSA strain. We hypothesized that the synergistic effect of biogenic AgNPs in combination with the antibiotic tigecycline could be attributed to the fact that both AgNPs and tigecycline targeted different bacterial cellular components. Tigecycline is a parenterally administered, bacteriostatic glycylcycline antibiotic that resembles tetracyclines structurally and has a greater (five-fold) binding affinity [95]. The main antibacterial mode of action of tigecycline is through the inhibition of the protein translation process of bacterial cells as the disruption of peptide chain elongation via reversible binding to a helical region (H34) on the 30S subunit of bacterial ribosomes [96]. Accordingly, tigecycline binding suppressed amino acid residues from being incorporated into peptide chains, which inhibits peptide synthesis and bacterial growth [97]. Based on the preceding, the supposed mechanism of AgNPs–tigecycline involves the action of AgNPs on the disruption of bacterial cell walls and membranes through the action of ROS, which also negatively disrupts bacterial DNA replication, while the tigecycline antibiotic suppresses bacterial protein translation and stops bacterial growth, leading to a synergistic shut down of biological activities of bacterial cells and induction of microbial cell death. The additive effect of linezolid antibiotic with biogenic AgNPs against MRSA strain was detected, whereas tigecycline antibiotic did not show any synergism with AgNPs against MRSA. Linezolid is the first antibacterial agent in the oxazolidinone class, which is used to treat nosocomial pneumonia, as well as mild and serious skin and soft tissue infections initiated by certain gram-positive bacteria [98]. In this context, linezolid inhibited bacterial protein synthesis by binding to rRNA on both the 50S and 30S ribosomal subunits, whereas AgNPs disrupted membrane permeability via ROS action and interrupted bacterial DNA replication, resulting in additive antibacterial activity when combined with linezolid. On the other hand, the biogenic AgNPs showed synergistic activity with the antifungal drug clotrimazole, disrupting the synthesis of ergosterol, the primary component of fungal cells’ membranes, depleting it, and replacing it with the aberrant sterol species 14-methylsterol, which then impairs membrane fluidity and permeability [99]. Additionally, the biogenic AgNPs exhibited an antifungal mode of action through interactions with DNA and protein, which disrupted these molecules and caused cell death, which then worked in a synergistic way with clotrimazole against *C. albicans* strain [100].

## 4. Conclusions

The biogenic AgNPs exhibited antibacterial and anticandidal activities against the tested strains. Ultrastructural investigation affirmed the deformation and distortion of bacterial and candidal cells treated with the biogenic AgNPs. The high efficiency of the biogenic AgNPs might be attributed to the small particle size of AgNPs, which enables them to penetrate the cell, and also to the action of capping biomolecules adsorbed over the surface of AgNPs. The biogenic AgNPs revealed the highest synergism with tigecycline against *A. baumannii,* which indicated the possible use of this combination for control of the multidrug-resistant pathogens colonizing the surfaces in ICUs and hospitals. In addition, tigecycline and linezolid exposed additive antibacterial efficacy with the bioinspired AgNPs against *E. coli* and MRSA strains, respectively. The biosynthesized AgNPs also exposed a synergistic activity with clotrimazole against *C. albicans*, indicating the possible usage of this combination to control the candidal colonization of medical equipment in ICUs and healthcare settings.

## Figures and Tables

**Table 1:** Functional groups of the biosynthesized AgNPs.

No.	Absorption Peak (cm^−1^)	Appearance	Functional Groups	Molecular Motion
1	3434.76	Strong, broad	Alcohols and phenols	O-H stretching
2	2920.38	Weak	Aldehydes	C–H stretching
3	1631.37	Medium	Carboxylic compounds or amides	C–O stretching or C–N bending
4	1106.76	Weak, broad	Aliphatic amines	C–N stretching
5	584.97	Weak, broad	Metal oxygen bond	Ag-O stretching

**Table 2:** Screening of antimicrobial efficiency of green synthesized AgNPs.

Microbial Strains	Inhibition Zone Diameter (mm)		
	AgNPs (200 μg/Disk)	Positive Control	Negative Control
MRSA	28.17 ± 0.21	20.56 ± 0.23 (TGC)	0.00 ± 0.00
*A. baumannii*	21.04 ± 0.19	20.63 ± 0.21 (TGC)	0.00 ± 0.00
*E. coli*	36.14 ± 0.67	22.67 ± 0.43 (TGC)	0.00 ± 0.00
*C. albicans*	18.16 ± 0.14	21.97 ± 0.52 (CLO)	0.00 ± 0.00

TGC: tigecycline, CLO: clotrimazole.

**Table 3:** Synergistic activity of different concentrations of biogenic AgNPs with commercial antibacterial and antifungal agents against the tested microbes.

The Tested Strains	Inhibition Zone Diameter (mm)				
	Antimicrobial Agent	AgNPs (MIC)	AgNPs (MIC) + Antimicrobial Agent	Synergism %	IFA
MRSA	29.18 ± 0.23 (LZD)	18.14 ± 0.18	32.76 ± 0.56	12.26%	0.3
*A. baumannii*	19.24 ± 0.16 (TGC)	11.23 ± 0.74	20.96 ± 0.42	8.93%	0.2
*E. coli*	35.24 ± 0.29 (TGC)	19.23 ± 0.37	38.78 ± 0.38	10.04%	0.2
*C. albicans*	20.98 ± 0.42 (CLO)	8.67 ± 0.67	22.78 ± 0.35	8.58%	0.2
	**Antimicrobial Agent**	**AgNPs** **(2-fold MIC)**	**AgNPs (2-fold MIC)** **+ Antimicrobial Agent**	**Synergism %**	**IFA**
MRSA	29.46 ± 0.11 (LZD)	24.14 ± 0.23	38.21 ± 0.36	29.70%	0.7
*A. baumannii*	19.67 ± 0.15 (TGC)	16.78 ± 0.46	29.14 ± 0.17	48.14%	1.2
*E. coli*	35.09 ± 0.29 (TGC)	25.56 ± 0.31	46.18 ± 0.56	31.60%	0.8
*C. albicans*	20.48 ± 0.54 (CLO)	14.97 ± 0.27	28.53 ± 0.48	39.30%	0.9

LZD: linezolid, TGC: tigecycline, CLO: clotrimazole.

**Table 4:** The fractional inhibitory concentration index (FICI) of the combined AgNPs and antibiotics.

The Tested Strains	Combined AgNPs + Antimicrobial Agent	FICI	Action
MRSA	AgNPs + TGC	1.75	No effect
	AgNPs + LZD	0.86	Additive
*A. baumannii*	AgNPs + TGC	0.38	Synergistic
*E. coli*	AgNPs + TGC	0.63	Additive
*C. albicans*	AgNPs + CLO	0.25	Synergistic

Synergistic effect ≤ 0.5; additive 0.5 to 1; no effect >1 to 4 and antagonistic >4. LZD: linezolid, TGC: tigecycline, CLO: clotrimazole.

## Data Availability

All required data are present in this file.
